# Influence of liver fibrosis stage on nevirapine plasma concentration in HIV-infected patients with chronic hepatitis C virus

**DOI:** 10.1186/1471-2334-14-S4-O17

**Published:** 2014-05-29

**Authors:** Gordana Dragovic, Colette Smith, Djordje Jevtovic, Mike Youle, David Back

**Affiliations:** 1 Institute of Pharmacology, Clinical Pharmacology and Toxicology, School of Medicine, University of Belgrade, Belgrade, Serbia; 2 Department of Primary Care and Population Sciences, Royal Free and University College Medical School, London, United Kingdom; 3 Institute of Infective and Tropical Disease “Dr Kosta Todorovic”, School of Medicine, University of Belgrade, Belgrade, Serbia; 4 Royal Free Centre for HIV Medicine, Royal Free and University College Medical School, University College London, London, United Kingdom; 5 Department of Pharmacology and Therapeutics, University of Liverpool, Liverpool, United Kingdom

## 

Hepatitis C virus (HCV) co-infection in HIV positive patients can affect the pharmacokinetics (PK) of antiretroviral drugs that are metabolized by the liver. Therefore, we determined plasma nevirapine (NVP) concentrations (C_trough_) in HIV+ patients with or without HCV co-infection.

This was a prospective study in patients receiving NVP (200 mg twice daily or 400 mg once daily) for at least 12 weeks as a part of antiretroviral regimen, at the HIV/AIDS Centre, Infective and Tropical Disease Clinic, Belgrade, Serbia. Written consent was obtained and the study was approved by the local ethics committee.

NVP plasma concentrations were measured by a validated HPLC-UV method at the University of Liverpool, UK. Statistical analysis was performed by SPSS software package.

27 patients (18 HIV mono-infected and 9 HCV/HIV co-infected patients) receiving NVP as part of their antiretroviral therapy were enrolled. The median age of the 27 patients was 43 years and all were Caucasian. In all patients HIV RNA was <50 copies/mL; median CD4+ T-cells count was 363 cells/cmm. Median NVP C_trough_ was 4826 ng/mL (2533-8718 ng/mL) in the HIV mono-infected group and 5810 ng/mL (4998-11783 ng/mL) in the HIV/HCV co-infected group, respectively. Compared to an individual with HIV mono-infection, those with HCV/HIV co-infection had a higher NVP trough level (95%CI +67 ng/mL, +2940 ng/mL; *P*=0.03). NVP plasma levels above the toxic threshold (8000 ng/mL) were more frequent in patients with liver cirrhosis than in those without (33% vs. 4%; *P* <0.001).

Despite the small numbers of patients included, this PK study has shown that NVP plasma levels are impaired in HIV-infected patients co-infected with HCV, especially in those with liver fibrosis.

